# Dietary diversity among school age children in Merawi town, Amhara region, Ethiopia, 2018: a community based cross-sectional study

**DOI:** 10.1186/s13690-019-0384-7

**Published:** 2020-01-06

**Authors:** Tilahun Tewabe, Amare Belachew, Yihun Miskir, Getnet Mekuria

**Affiliations:** 1 College of Medicine and Health science, Bahir Dar University, Bahir Dar, Ethiopia; 2 Bahir Dar University, Bahir Dar University Institute of Technology, Bahir Dar, Ethiopia

**Keywords:** Dietary diversity, Prevalence, Household food security, School-age children, Merawi town, Ethiopia

## Abstract

**Background:**

Malnutrition with its constituents of protein energy malnutrition and micro-nutrient deficiencies continues to be a major health burden in low and middle-income countries. To end all forms of malnutrition, we need to address poverty, which is associated with the insecure supply of food and diversified nutrition. The objective of this study was to determine the level of dietary diversity and household food security among urban school-age children in Merawi town, Ethiopia.

**Methods:**

A community based cross-sectional study was conducted in Merawi town among 422 households having school age children from April 1 to June 15, 2018. The association between dietary diversity and determinants was assessed using binary logistic regression analysis. Socio-demographic, maternal and child related variables; food security and diversity determinants were studied.

**Results:**

The overall level of good dietary diversity was 91.7%, i.e.; 8.3% had a low, 59.1% had a good, 32.6% had better dietary diversity, respectively. Most households (95.2%) were secured with food access. The factors associated with good dietary diversity were the age of the child [AOR = 0.31 (0.14, 0.70)], and access to information [AOR = 3.18 (1.07,9.47)].

**Conclusion:**

The prevalence of good dietary diversity was relatively high. Among different socio-cultural and economic factors studied, age of the child and access to information were the factors associated with dietary diversity. Increasing maternal and child awareness towards good dietary diversity practices through the mass media (radio and Television) and working with mothers with early school-age children to improve dietary diversity are recommended.

## Background

Malnutrition results from chronic nutritional and frequently emotional deprivation by careers because of poor understanding, poverty or family problems making them unable to provide the child with the nutrition and care he or she requires [1]. Malnutrition constituents protein energy malnutrition and micro-nutrient deficiencies and it is a major health burden in developing countries. It is globally the most important risk factor for illness and death in children, with hundreds of millions of young children are affected [2].

The 2011 Ethiopian Demographic and Health Survey (EDHS) illustrated the percentage of children who were stunted was 44% and 29% of all children were underweight [3]. Globally, it is estimated that nearly 20 million children are severely malnourished [4].

This is because in most countries with diverse agro-climatic conditions, children consume a monotonous diet which has adverse consequences for their nutritional status [5]**.** Children with good dietary diversity were associated with normal body weight [6] and the optimal level of dietary diversity were related with increased intake of micro-nutrients [7] and decreased level of stunting [8]**.**

Dietary diversity was a promising indicator of food security in a study done in five municipalities in Greater Sekhukhune, Limpopo Province, South Africa [9]**.** A multitude of environmental, demographic, economic, infrastructural and social factors were causing seasonal food insecurity. The identified causes were drought, erratic rainfall patterns, livestock and crop diseases, dependency on a single season harvest per year [10]**.** In China the positive predictors of dietary diversity were linked with those living in the urban environment, while high dietary diversity were associated with a decreased risk of anemia [11]**.** It revealed dietary diversity varied with age and place of residence; the older and the one’s living in rural areas have poorer dietary diversity [12]**.**

The proportion of children who consumed minimum acceptable dietary diversity was 43.2% in wolayita sodo region [13]**,** 16.2% in South Gonder [14]**,** 13.6% in Dejen [15]**,** 13% in Sinan woreda [16]**,** 34.3% in Mirab Abaya woreda Southern Ethiopia [17]**.** Regarding the level of food insecurity; 48% in Amhara Ethiopia [18]**,** 53% in Gojjam [19], 78.1% in Butajira hospital [20], 63.0% in Jimma [21], 58.16% in Addis Ababa [22] and 58% in East Hararghe zone [23]. The proportion of food security was higher during harvest season (31.3%) than during the rainy season (19.9%) [24]**.**

At the community level, access to irrigation infrastructure strengthened food security, and was the most transformative difference between the communities [25]**.** Over the past 20 years, Ethiopia has made significant progress in improving health, nutrition, education, and in other human development indicators [26]**.** Studies showed household decisions about the adoption of agricultural technology and their food security situations were strongly and positively interdependent [27]**.** In Ethiopia food security is mostly depended on rain based agriculture [28]**.** Having this appropriate child feeding is a means for achieving the sustainable development goals (SDG) pertaining to child health, particularly for goal 2.2 by 2030 to end malnutrition and to achieve the 2025 internationally agreed set points to eliminate stunting and wasting [29].

Though good dietary diversity and appropriate feeding is known to enhance the growth and development of children, studies conducted in other areas are inconclusive and inconsistent beyond this there is little information available on dietary diversity and household food security among children in the study area. Identifying factors associated dietary diversity will be an important aspect of intervention to improve childhood nutrition with local available resources to the local health system, non-governmental bodies, stakeholders and for the nation. The study will support the local community by directly increasing their knowledge on child health promotion, nutrition and illness prevention. Childhood nutrition is one among the most priority agendas of the regional health bureau to decrease childhood malnutrition and related conditions and this study will be an asset for substantial improvement in child health. Therefore, the purpose of this study was to assess the level dietary diversity and household food security among urban school-age children in Merawi town, Northwest Ethiopia.

## Methods

### Study settings and participants

A community based cross-sectional study was conducted from April 01 to June 15, 2018. The study was conducted in Merawi town which is located in Amhara regional state, Ethiopia and 535 km away from Addis Ababa. It is 30 km far from Bahir Dar. The town has 3 kebeles (part of the town divided for administrative purposes). Merawi is estimated to have a total population of 35,541 of whom 18,479 were males and 17,062 were females. There is one public hospital and one health center in the town [30].

The sample size was calculated using single population proportion formula $\mathrm{n}=\frac{z_{a/2}^2\times p\left(1-P\right)\;}{d^2}$ by considering the following assumptions: P (proportion of dietary diversity among school-age children) = 50% to get the maximum sample size to represent the community, margin of error (d) = 5%, confidence level (CL) = 95% and after considering 10% non-response rate the final sample size was 422. To select study participants, households having school-age children were selected using simple random sampling technique (lottery method).

### Sampling procedure

All three kebeles of the town were included in the study. Then, sample from each kebeles was determined using proportional allocation to size. Finally, study subjects were selected using lottery method. If over one child with a similar age group was found in the same house, then the youngest child was selected. If the eligible child was absent from the house at the time of data collection, revisit was done again and if they were absent at the second visit it was considered to be non-respondent.

### Data collection tools and procedure

The data was collected by four trained nurses using structured interviewer administered questionnaire, which was adopted and modified from the Food and Agriculture Organization of the United Nations (FAO)/ Food and Nutrition Technical Assistance (FANTA) Guidelines for measuring Household and Individual Dietary Diversity tools and from previous research done on a similar topic [12–16, 19–23]. The questionnaire was first prepared in English then translated to Amharic local language for data collection and back translated for consistency. It comprised of four parts: socio-demographic characteristics, maternal and child health utilization, dietary diversity and food security questionnaire were incorporated. For measuring dietary diversity and food security the questionnaire were designed to have had ‘yes’ or ‘no’ options, and for others multiple-choice questions were used which was further categorized for analysis in binary logistic regression analysis. Data collectors were trained for a day about collection techniques and procedures before the actual time of collection. The data was collected by face-to-face interview of mothers with their school-age children: socio-demographic variables and environmental characteristics, maternal/child behaviors and dietary diversity and food security data were taken.

### Data quality assurance

All activities were done with the agreement of the principal investigator to get qualified data and the questionnaire was pretested on 5% of sample size in the nearby city called Meshente and some modifications like spelling and appropriate selection of words was done. Training was given in each module of the questionnaire on how to ask questions and code answers. To ensure data quality, completeness, accuracy and consistency all collected data was checked every day by the investigator and supervisor during the entire data collection period. Any quest related to clarity, ambiguity, incompleteness, misunderstanding, was solved on the following day before the next day activities.

### Variables of the study

#### Dependent variable

Dietary diversity.

#### Independent variable

Child related variables; age, sex, birth interval, birth size, prelacteal feeding, immunizations, breastfeeding and weaning practices, late initiation of complementary feeding, inadequate feeding, parent related variables; educational status, place of delivery, family planning, maternal health seeking behavior, number of children, household/social variables; food availability, access to information, misconception, access to iodized salts, sanitation, agricultural patterns, drought, floods, wars, food scarcities.

### Operational definition

A child is considered having a good diet diversity when he/she eats at least four types of food groups per day (within 24 h).

School-age children refers to a child with age ranges between 6 and 12 years.

### Data analysis

Data was entered and cleaned using Epi info version 7 and exported to SPSS software version 21 for analysis. Both descriptive (frequency tables) and inferential statistics were used to present the data. Binary logistic regression was performed to examine the association of each independent variable on the outcome variable dietary diversity. First variables tested to examine the association between each independent variable and the outcome variable, dietary diversity by using bivariate analysis and variables which showed association in the bivariate analysis were included in the final multivariate logistic regression analysis. The results were presented with crude odds ratios (COR) and adjusted odds ratios (AOR) and their respective 95% confidence intervals. Statistical significance was declared at *P* value < 0.05.

## Results

### Socio demographic characteristics

From all eligible study subjects about 396 participated in this study, which made a response rate of 93.8%. Almost half (50.8%) of the children were males, below half (47%) were between 6 and 8 years of age and almost one third (30.3%) were first in birth order. Regarding the mother; most (96.5%) were between 18 and 45 years, more than half (59.6%) of mothers had a family member of five and above, less than (46.5%) of mothers have 3 to 4 children and almost two third (65.2%) mothers were orthodox christian followers. Concerning to educational status of mothers, above half (55.8%) of mothers were educated. Less than half (42.2%) of mothers were employed, and most (82.3%) mothers were married. Pertaining to husband education, near two-thirds (64.7%) were educated and lower than half (46.9%) were employed. Almost one in ten (12.6%) households had their own farming land. Majority households (93.7%) had access to information from different sources (Table 1).

**Table 1:** Socio-demographic distribution of school age children in Merawi town, North West, Ethiopia, 2018 (*n* = 396)

Variable	Response	Frequency	Percent
Sex of the child	Male	201	50.8
	Female	195	49.2
Age of the child	6–8 years	186	47.0
	9–10 years	134	33.8
	11–12 years	76	19.2
Number of children in the household	1–2	179	45.2
	3–4	184	46.5
	Five and above	33	8.3
Family size	1–2	10	2.5
	3–4	150	37.9
	5+	236	59.6
Birth order of the child	First	120	30.3
	Second and above	276	69.7
Age of the mother	18–45	381	96.2
	46–60	15	3.8
Mother’s religion	Orthodox christian	258	65.2
	Muslim	100	25.3
	Protestant	38	9.6
Mother’s ethnicity	Amhra	395	99.7
	Others	1	0.3
Mother’s education	Educated	221	55.8
	Uneducated	175	44.2
Mother’s occupation	Employed	167	42.2
	Farmer	13	3.3
	Unemployed	216	54.5
Mother’s marital status	Married	326	82.3
	Unmarried	70	17.7
Husband level education	Educated	233	64.7
	Uneducated	127	35.3
husband’s occupation	Employed	169	46.9
	Unemployed	191	53.1
Ownership to farming land	Yes	50	12.6
	No	346	87.4
Irrigation user	Yes	30	7.6
	No	366	92.4
Average monthly income (Ethiopian birr)	< 1000	40	10.1
	1001–2000	84	21.2
	> 2001	272	68.7
Access to information	Yes	370	93.4
	No	26	6.6

### Maternal and health related characteristics

About 333 (84.1%) mothers used family planning methods. Regarding children; most (86.4%) of them were on school, about half (50.3%) were grade three and above, slightly above one tenth (13.9%) were engaged in work among them more than half (55.4%) worked for over 3 hours. Most (75%) of children had a history of illness and about one third (37.6%) of them took additional feeding during an illness. Majority (96.5%) of children eat breakfast regularly and most (85.1%) children eat four and above per day. Most (90.9%) mothers had access to child nutrition education (Table 2).

**Table 2:** Maternal and child related characteristics distribution of school age children in Merawi town, North West, Ethiopia, 2018 (*n* = 396)

Variable	Response	Frequency	Percent
Ever used of family planning methods	Yes	333	84.1
	No	63	15.9
Is the child on school	Yes	342	86.4
	No	54	13.6
Level/ grade of education	1–2 grade	172	49.7
	Grade three above	174	50.3
Is the child engaged in work	Yes	55	13.9
	No	341	86.1
If engaged in work for how many hours (*n* = 55)	≤3 h	30	54.5
	Above 3 hours	25	45.4
does the child get feeding if he/she engages in work for more than 3 h (*n* = 55)	Yes	20	36.4
	No	35	63.6
Does the child has history of illness	Yes	297	75.0
	No	99	25.0
What type feeding do you give for the child during illnesses	Regular family dish	247	62.4
	Additional feeding	149	37.6
Does the child eat breakfast regularly	Yes	382	96.5
	No	14	3.5
Frequency of eating per day	Four and above	336	84.6
	1–3	60	15.2
Access to child nutrition education	Yes	360	90.9
	No	36	9.1

### Household food security and dietary diversity

Most households (95.2%) were secured with food access scales. In the past 4 weeks; 3.3% were worried about household food shortage, 3.0% could not eat the kinds of foods they preferred, 2.5% eat a limited variety of foods, 1.3% go to sleep at night hungry and 1.8% go a whole day and night eating nothing.

With regard to dietary diversity; 8.3% had a low, 59.1% had a good, 32.6% had better dietary diversity, respectively. Concerning to food types: 87.4% took any local foods; bread, rice noodles, biscuits, or any other foods made from millet, sorghum, maize, rice, wheat, or any other locally available grain, 65.2% eat any potatoes, yams, manioc, cassava or any other foods made from roots or tubers, 34.8% feed vegetables, 29.8% took fruits, 36.4% eat eggs, 72.5% took foods made with oil, fat, or butter, 53.5% took any other foods, such as condiments, coffee, tea (Table 3).

**Table 3:** Household food security and dietary diversity distribution of school age children in Merawi town, North West, Ethiopia, 2018 (*n* = 396)

	Response	Frequency	Percent
Food security questions items			
In the past 4 weeks, did you worry that your household would not have enough food	Yes	13	3.3
	No	383	96.7
In the past 4 weeks, were you or any household member not able to eat the kinds of foods you preferred because of a lack of resources	Yes	12	3.0
	No	384	97.0
In the past 4 weeks, did you or any household member have to eat a limited variety of foods due to a lack of resources	Yes	10	2.5
	No	386	97.5
In the past 4 weeks, did you or any household member have to eat some foods that you really did not want to eat because of a lack of resources to obtain other types of food	Yes	8	2.0
	No	388	98.0
In the past 4 weeks, did you or any household member have to eat a smaller meal than you felt you needed because there was not enough food	Yes	9	2.3
	No	387	97.7
In the past 4 weeks, did you or any household member have to eat fewer meals in a day because there was not enough food	Yes	8	2.0
	No	388	98.0
In the past 4 weeks, was there ever no food to eat of any kind in your household because of lack of resources to get food	Yes	5	1.3
	No	391	98.7
In the past 4 weeks, did you or any household member go to sleep at night hungry because there was not enough food	Yes	5	1.3
	No	391	98.7
In the past 4 weeks, did you or any household member go a whole day and night without eating anything because there was not enough food	Yes	7	1.8
	No	389	98.2
Dietary Diversity questions items			
Any local foods, bread, rice noodles, biscuits, or any other foods made from millet, sorghum, maize, rice, wheat, or any other locally available grain	Yes	346	87.4
	No	50	12.6
Any potatoes, yams, manioc, cassava or any other foods made from roots or tubers	Yes	258	65.2
	No	138	34.8
Any vegetables	Yes	138	34.8
	No	258	65.2
Any fruits	Yes	118	29.8
	No	278	70.2
Any beef, pork, lamb, goat, rabbit wild game, chicken, duck, or other birds, liver, kidney, heart, or other organ meats	Yes	183	46.2
	No	213	53.8
Any eggs	Yes	144	36.4
	No	252	63.6
Any fresh or dried fish or shellfish	Yes	33	8.3
	No	363	91.7
Any foods made from beans, peas, lentils, or nuts	Yes	213	53.8
	No	183	46.2
Any cheese, yogurt, milk or other milk products	Yes	195	49.2
	No	201	50.8
Any foods made with oil, fat, or butter	Yes	287	72.5
	No	119	27.5
Any sugar or honey	Yes	181	45.7
	No	215	54.3
Any other foods, such as condiments, coffee, tea	Yes	212	53.5
	No	184	46.5

### Factors associated with dietary diversity

The independent predictors dietary diversity were education of mothers, marital status, maternal occupation, family size, age of the child, access to information and household food security.

The final predictors of dietary diversity were the age of the child and access to information.

Age of the child was significantly associated with the prevalence of higher dietary diversity. A child with in age group of 6–8 years were almost 70% lower to have good dietary diversity than a child with in the age group 9–12 years old [AOR = 0.31 (0.14, 0.70)].

In this study, access to information was significantly associated with dietary diversity. Mothers of a child having better access to information were almost three times higher to have good dietary diversity than a mother of a child had not got access to information regarding child feeding practices [AOR = 3.18 (1.07,9.47)] (Table 4).

**Table 4:** Distribution of dietary diversity among urban school age children in Merawi, North West Ethiopia, 2018 (*n* = 396)

Variables	dietary diversity				
		Yes	No	COR (95% CL)	AOR(95% CL)
Education	Educated	210	11	3.034 (1.396, 1.396)	
	Uneducated	153	22	1	
Marital status	Married	296	30	.459 (0.136, 1.550)	
	Not Married	67	3	1	
Maternal occupation	Employed	158	9	2.327 (1.018, 5.318)	
	Unemployed	205	24	1	
Family size	Less than or equal four	151	9	1.812 (0.815, 4.025)	
	Equal or greater five	212	24	1	
Age of the child	6–8			0.373 (0.172, 0.809)	**0.31 (0.14, 0.70)**
	9–12			1	**1**
Access to information	Yes	343	27	3.969 (1.467,10.742)	**3.18 (1.07,9.47)**
	No	20	6	1	**1**
Household food security	Insecure	16	3	.444 (.122, 1.615)	
	Secured	347	30	1	

1 = reference, *COR* Crude odds ratio, *AOR* adjusted odds ratio and * = significant variable

## Discussion

In this study, the level of dietary diversity was relatively high, 33% of the children had a high dietary diversity and 59 and 8% had a good and low dietary diversity respectively.. This study is better than researches conducted based on EDHS study 10.8% [31], in Sinan woreda 13% [16], in Dejen woreda 13.6% [15], Gonder Zone 16.2% [14], in Mirab Abaya woreda Southern Ethiopia 34.3% [17] and in wolayita sodo region 43.2% [13]**.** This may be because of the socio-cultural, economic, geographical differences between the study area and the referenced places. While in other areas peoples are commonly practicing monotonous feeding in relation to the agricultural patterns and seasons. Dietary diversity is better during a summer season. It is because of more fruits, vegetables, potatoes, yams, manioc, cassava or any other foods made from roots or tubers in summer season, while winter seasons is better for other foods. Next, the fasting rules of the Ethiopian Orthodox Church may affect the dietary diversity negatively. The number of fasting days are huge, i.e. over 210 days per year and in most situations children fast by the age of seven (during school-age period) [32]. This ultimately decreases food intakes like meat, egg, milk.

Most households (95.2%) were food secured with food access scales. In the past 4 weeks only 3.3% were worried about the shortage of feeding. This finding is significantly higher than studies done in Amhara Ethiopia where about 48% households were food insecure [18], Fedis Woreda East Hararghe zone, Oromia region, Ethiopia 58% of households were food insecure [23], in Addis Ababa Ethiopia 58.16% households were below food insecurity line [22]**,** In Jimma 63.0% PLWHA were food insecure [21], food insecurity among people living with HIV/AIDS (PLWHA) receiving highly active anti-retroviral therapy (HAART) at Butajira hospital was 78.1% [20] and in Gojjam Ethiopia food insecurity among podoconiosis patients household was 83.7% [19]**.** This may be because the study period was a spring where in most families there is no problem of food insecurity which similar with the above studies where all were conducted in the winter and spring seasons. The proportion of food secured households was higher during harvest season (31.3%) than a rainy season (19.9%) [24], since it is the post-harvest season in Ethiopia where in most households there is a problem in the availability foods.

In this study, the prevalence of dietary diversity was affected by different factors. Among them the age of the child was significantly associated with the prevalence of dietary diversity. A child with in age group of 6–8 years had a lower dietary diversity than elder school-age children i.e., between 9 and 12 years**.** This result is consistent with studies conducted in Wolaita Ethiopia [13], but it is inconsistent with findings in China [12] . This may be due to the fact that as the age of the child increases their habit of food choices will increase because of formal and informal learning like influences and discussions with peers, environmental scanning and school based learning.

Access to information was the determinant factors for dietary diversity. Mothers with children having better access to information were almost three times higher to have good dietary diversity than their counterparts***.*** This is comparable to studies based EDHS [31], in Gonder [14], in Dejen district, Northwest Ethiopia [15]. Mass media are critical in disseminating public health information, improving health knowledge and changing health behaviors. However, most of the mass media public health interventions do not sufficiently engage the local people; they are externally determined. Because of this, very little is known about the effects of locally instigated mass media promotion. Media campaigns on diet diversity showed that most of the interventions had short-term effects to the extent that demand the community to increase dietary diversity and decease the level of malnutrition.

As strength the study identified determinants of dietary diversity which could be an important aspect of increasing diet diversity of school-age children. A limitation of the study is related to the fact that the data were only collected during the spring season. This may have induced a recall bias related to the dietary intake information and its seasonal variations as well as related to the health care utilization of the mother and the child.

## Conclusion

The prevalence of dietary diversity was generally high in the study area. Among different socio-cultural and economic factors studied, only the age of the child and access to information were the determinant factors of good dietary diversity. Increasing maternal and child awareness towards dietary diversity through media, working with mothers with early school-age children to improve dietary diversity were recommended.

## Data Availability

The date of this study can’t be shared publically due to presence of sensitive (confidential) participants’ information.

## Abbreviations

AOR: Adjusted odds ratio
CL: Confidence level
COR: Crude odds ratio
EDHS: Ethiopian demographic health survey
SD: Standard deviation
UNICEF: United Nation Children Emergency Fund
WHO: World Health Organization
